# “In vitro comparison of the adsorption of inflammatory mediators by blood purification devices”: a misleading article for clinical practice?

**DOI:** 10.1186/s40635-018-0214-1

**Published:** 2019-01-09

**Authors:** Marco Feri

**Affiliations:** Department of Intensive Care, Perioperative Medicine and Antalgic Therapy, Azienda USL Sud Est, San Donato Hospital, Via Pietro Nenni, 52100 Arezzo, Italy

## To the Editor,

I find this article from Malard et al. very interesting, since it aims to analyze the effect of three blood purification devices—CytoSorb, Toraymyxin, and oXiris—in removing toxic molecules involved in septic shock [1]. However, the study shows some limitations that could lead to the incorrect clinical use of these sorbents.

The experiments were performed for 2 h using 500 ml plasma solutions from healthy volunteers, which were pre-incubated with pathologic quantities of inflammatory mediators:

1. In clinical practice, all three devices work with whole blood, whose components are different from those of plasma.
2. The volume of plasma used during all tests (500 ml) is very scant, given that examined devices work with a blood volume of about 5 l and the analyzed molecules continuously regenerate in clinical setting. This scarce volume of the solution can seriously affect the results of this study.
3. The concentration of inflammatory mediators is low. This study, for example, reports IL-6 concentration of 1500 pg/ml, but septic patients show much higher IL-6 levels [2, 3]. Furthermore, inflammatory mediator values in blood always change, since their kinetics are not constant.
4. All experiment duration is equal to 2 h, which is not the actual application time of CytoSorb and oXiris, since the first one can be used for 24 h and the second one for a maximum of 72 h. Adsorption treatment duration is essential in defining the saturation of each device.

Performing the experiments with a scant 500 ml plasma solution with low and not regenerated concentrations of inflammatory mediators for only 2 h necessarily results in total removal of the incubated substances by the three devices. After that, there is nothing left to be adsorbed, and it is not possible to assess the saturation time of the evaluated devices, so it is not possible to understand which one effectively saturates and which one still has adsorptive capabilities.

Moreover, since all three devices work in adsorbing specific molecules, a worth knowing parameter—not mentioned in this article—would be their surface area, essential to define their adsorptive capabilities and their saturation. Oxiris has a surface area of 1.5 m^2^, while the surface areas of Toraymyxin and CytoSorb run into thousands of square meters. This experimental setting does not point out how the three sorbents saturate, so it appears that they are all equally efficient in adsorbing their target molecules. This is probably true if you consider the only 2-h-long in vitro experiments in a small closed circuit, but it is not like that in clinical practice, where the devices work in a varying open system for a longer time and with higher and always changing target compound values.

A further analysis would be necessary to evaluate these sorbents and define the real differences among them.
