# Supplementary material for: “In vitro comparison of the adsorption of inflammatory mediators by blood purification devices”: a misleading article for clinical practice?
Source: Intensive Care Med Exp. 2019 Jan 9;7:5. doi: 10.1186/s40635-018-0214-1 (PMC6326912; doi:10.1186/s40635-018-0214-1)
Supplement: Related texts

# Reply: Response from the authors

**Authors:** Benjamin Malard, John A. Kellum

We thank Dr. Feri for his interest in our paper. The intention of our manuscript was to document mechanistically the removal spectrum of different blood purification devices that may be used in the ICU according to their respective intended use. By design, this in vitro study cannot properly simulate the extremely complex patient conditions from which the intended patient population may suffer. Those limitations are clearly stated in the manuscript to avoid any misleading interpretation from a clinical practice, referring notably to “First, as an in vitro study the outcomes observed here may not be representative of the clinical setting.”

Secondly, from a methodological perspective, human plasma is preferred to whole human blood because this permitted an identical setup for each experiment with the intention to interpret the observations in a relative manner. This limitation was also clearly acknowledged in the limitation section. Furthermore, each mediator selection is based on available literature on sepsis pathogenesis. Supplemental data (Table S1) clearly supports the pathological mediator concentrations selected, extrapolated to a human plasma volume of 2.5 l.

We agree that the kinetics of inflammatory mediators in the blood are not constant. For that reason, the limitation section clearly states that “our data can only be used to comment on the adsorption of these mediators at these concentrations.”

Dr. Feri also suggests that our methodology cannot properly compare these devices in accordance with their clinical duration and that the oXiris device may have reduced adsorptive capacities in relation to a lower surface area. To the question of the experiment duration, a 2-h session is the average treatment duration reported with the Toraymyxin cartridge in recent large-scale clinical studies [4–6]. CytoSorb may be used 6 h per day according its instruction for use (not 24 h as mentioned in the letter) [7]. The question of the experimental duration is interesting, and therefore, a calculation of clearance was proposed (Fig. 4), without any ambiguity on the respective instantaneous removal performances of each device.

To the question of the surface area, in the oXiris membrane, the microstructure and the chemical composition of the AN69 polymer offer a unique context for bulk adsorption of middle molecular weight proteins, the high water content of the hydrogel making the polymer chains easily accessible [8]. This implies that the surface area accessible for adsorption is considerably increased, far above the surface area that is at the blood membrane interface (i.e., 1.5 m^2^ for oXiris). Sakai et al. have notably proposed a model to simulate hydrogel total surface accessible for adsorption [9]. In case of oXiris, the total theoretical surface area accessible for adsorption would basically consist of approx. 17,000 m^2^. Of note, Nishida et al. have shown that the AN69 hydrogel membrane does not reach saturation at supra pathological quantities of HMGB-1 (seven successive pathological injection of 100 μg) [10], further illustrating this point.

The authors acknowledge that it would be interesting to determine the total amount adsorbed for each device (which was not the intent of our study) and that this preliminary work would warranty further investigations. However, our study conclusion does not provide any misleading information on how to use clinically the investigated devices and clearly provides associated limitations to the disclosed results.
